# Chinese woodchucks with different susceptibility to WHV infection differ in their genetic background exemplified by cytochrome B and MHC-DRB molecules

**DOI:** 10.1186/s12985-018-1010-y

**Published:** 2018-06-18

**Authors:** Bin Zhu, Zhenni Zhu, Junzhong Wang, Shunmei Huang, Fanghui Li, Lu Wang, Yanan Liu, Qi Yan, Shunchang Zhou, Mengji Lu, Dongliang Yang, Baoju Wang

**Affiliations:** 1 Department of Infectious Diseases, Union Hospital, Tongji Medical College, Huazhong University of Science and Technology, 1277# Jiefang Avenue, Wuhan, 430022 China; 2 Institute of Virology, University of Duisburg-Essen, Essen, Germany; 3 Laboratory Animal Center, Huazhong University of Science and Technology, Wuhan, China; 4 Department of Pediatrics, Maternal and Child Health Hospital of Hubei Province (Women and Children’s Hospital of Hubei Province), Wuhan, China

**Keywords:** Chinese woodchuck, Cytochrome B, Major histocompatibility complex class II DRB gene, Hepatitis B virus, Woodchuck hepatitis virus

## Abstract

**Background:**

Chinese woodchucks (*M. himalayana*) were recently found to be susceptible to woodchuck hepatitis virus (WHV) infection. In this study, we aimed to determine the susceptibility to WHV infection of *M. himalayana* from different areas and their association with the animal genetic background exemplified by cytochrome B and MHC-DRB molecules.

**Methods:**

Animals from four different areas in Qinghai province were inoculated with WHV59 strains. The virological markers including WHV surface antigen (WHsAg), WHV core antibody (WHcAb), and WHV DNA in serum were measured by ELISA and Real-time PCR, respectively. The sequences of cytochrome B gene and MHC-DRB molecules were obtained and sorted with Clustalx software. The nucleotide variation sites were identified using MEGA5 software.

**Results:**

The animals from four different areas had different susceptibility to WHV infection. Animals from TR and TD areas had a high level of long-lasting viremia, while those from GD and WL areas had a low level of transient viremia after WHV inoculation. All of the animals belong to the same subspecies *M. himalayana robusta* identified by cytochrome B gene sequences. Based on their nucleotide variation pattern, 8 alleles of cytochrome B gene were identified, and 7 MHC-DRB alleles were identified. Allele A of cytochrome B and Allele Mamo-DRB1*02 of MHC-DRB was found to be frequent in animals from TR and TD areas, while Allele H of cytochrome B and Allele Mamo-DRB1*07 of MHC-DRB was predominant in animals from GD and WL areas.

**Conclusion:**

Chinese woodchucks from different areas differed in their susceptibility to WHV infection, though they belong to the same subspecies *M. himalayana robusta*. The genetic background exemplified by cytochrome B and MHC-DRB differed in Chinese woodchucks with different susceptibility to WHV infection.

**Electronic supplementary material:**

The online version of this article (10.1186/s12985-018-1010-y) contains supplementary material, which is available to authorized users.

## Introduction

Hepatitis B virus (HBV) is the prototype of the hepadnavirus family and causes acute or chronic infection. An estimated 2 billion people are infected with HBV worldwide, and more than 240 million people are HBV carriers and are at high risk for developing hepatic decompensation, cirrhosis, and hepatocellular carcinoma [1]. Currently, interferon alpha and nucleoside/nucleotide analogues, e.g. entecavir and tenofovir, are approved for the treatment of chronic hepatitis B. Since interferon alpha leads to a sustained antiviral response in only one third of patients, and nucleoside/nucleotide analogues cannot completely eradicate the virus, HBV infection remains a serious public health issue in Asia and the rest of the world [2].

Woodchuck hepatitis virus (WHV) is a naturally occurring hepadnavirus of the American woodchuck (*Marmota monax*), and was initially discovered in 1978 at the Penrose Zoo in Philadelphia in a colony of woodchucks where high rates of chronic hepatitis and HCC had been observed [3]. Since WHV infection in American woodchuck resembles human HBV infections in its major virological, pathological and immunological features, the American woodchuck model is widely used to study HBV pathogenesis and evaluate the antiviral drugs or the therapeutic vaccines [4–6]. Due to the high incidence of chronic HBV infection in China [7], there is a strong interest to develop an animal model with woodchuck species available in China. The Chinese woodchuck species are mainly located in the north-west of China, are phylogenetically related to the American woodchucks, and can be transmitted by WHV in young animals [8–11]. Though there are no solid evidences for the naturally occurring WHV infection in Chinese woodchuck species, we recently found that different Chinese woodchuck species have different susceptibility to the experimental WHV infection [12]. While *M. himalayana* was fully susceptible to WHV, *M. baibacina* showed no sign of WHV infection after inoculation with the same WHV stock. *M. bobak* seems to show a limited susceptibility to WHV infection.

WHV infection in American woodchuck was found to be endemic in areas of the mid-Atlantic states, but to be apparently absent from populations in New York and much of New England [13]. The New York and Delaware woodchucks are considered as the northern (*M. monax rufescens*) and southern (*M. monax monax*) subspecies, respectively, thereafter Tyler et al. speculated that the subspecies may be responsible for the different rate of WHV infection in American woodchuck [14]. While the possible difference in the susceptibility to WHV infection among *M himalayana* from different areas is still unknown.

The vertebrate mitochondrial genome presents certain features, such as compact organization, maternal transmission, relatively small size, hundreds to thousands of copies per cell, rapid evolution, and a lack of or much reduced recombination [15] . Therefore, partial or complete sequences of the mitochondrial genome, in particular the sequences of the mitochondrial cytochrome B, have been used extensively in studies on population genetics, phylogeny and phylogeography in *Rodentia* [16].

The major histocompatibility complex (MHC) plays a crucial role in the adaptive immune response of vertebrates. The MHC is a multigene family encompassing classical class I and class II genes, as well as nonclassical MHC genes and pseudogenes. Human leukocyte antigen (HLA) is an integral component of the immune response on which the majority of host genetic studies have concentrated. Many different HLA alleles have been demonstrated to play a role in HBV infection [17, 18]. It has been reported that certain HLA DRB alleles correlate with the outcome of HBV infection [19].

In this study, we trapped 48 *M. himalayana* from four areas, and found they have different susceptibilities to WHV infection. We aimed to determine the association between WHV infection and the genetic background of *M himalayana* exemplified by cytochrome B and MHC-DRB molecules.

## Methods

### Animals

Chinese woodchucks were captured in the Tongren (TR), Wulan (WL), Tongde (TD), and Guide (GD) counties, Qinghai province, China, and provided by the Qinghai Institute for Endemic Disease Control and Prevention (Xining, Qinghai, China). Tests for WHV core antibody (WHcAb), surface antigen (WHsAg) and antibody (WHsAb), and WHV DNA were conducted to exclude previous exposure to WHV. The general information of the Chinese woodchucks used in this study is shown in Additional file 1: Table S1. Experiments were conducted in accordance with the Guide for the Care and Use of Laboratory Animals (National Academy Press, revised 1996) and were reviewed and approved by the local Animal Care and Use Committees (Animal Ethics Committee of Tongji Medical College, Huazhong University of Science & Technology, China). (IACUC Number: S659).

### Experimental WHV infection

Four animals from each area were inoculated with WHV59 strains, provided by Dr. Mengji Lu from the Institute of Virology, University of Duisburg-Essen. Blood was collected every two weeks, and the sera were isolated and frozen at − 20 °C. The serum samples were diluted 1:10 in phosphate buffered saline, and WHsAg and WHcAb were detected by ELISA as described previously [12, 20]. The results for WHsAg are presented as S/N values = OD of sample / OD of negative control. The results for WHcAb are presented as the percentage of inhibition = [(OD of negative control – OD of sample) / (OD of negative control) × 100]. Viral DNA was isolated from serum using the E.Z.N.A® Viral DNA Kit (Omega, Doraville, GA, USA). Real-time polymerase chain reaction (PCR) was performed with a SYBR® Green Real-time PCR Master Mix (Toyobo, Osaka, Japan), according to the manufacturer’s instructions. The PCR primers used to amplify viral DNA were QP1 and QP2, as described previously [12, 20]. Plasmids containing the WHV full-length DNA were serially diluted and used as standard. The detection limit of this assay was at 10^2^ WHV genome equivalents per reaction.

### DNA and RNA isolation

Whole blood was collected from Chinese woodchucks and total DNA were isolated using EZNA Blood DNA kit (Omega Biotek, Doraville, GA). RNA was isolated from the whole blood by using RNAiso plus total RNA extraction reagent according to the manufacturer’s instructions (TaKaRa, Dalian, China).

### PCR, RT PCR and sequencing

The PCR Primers cytB-1/2 were designed according to the cytochrome B sequence of M. Himalayan (AY143928). The RT-PCR Primers DRB-1 and DRB-2 were designed according to DRB sequence from *Spermophilus tridecemlineatus* (XM_005338936.1). Primers were designed by using Primer Premier 5 software and Oligo7 software. All the Primers were synthesized in a local commercial company (Invitrogen Life Technologies Corporation, Shanghai) and are shown in Additional file 1: Table S2. Complementary DNA was synthesized from 1 μg of purified total RNA by using a reverse transcription (RT-PCR) system (Prime Script RT Reagent Kit; TaKaRa, Dalian, China) according to the manufacturer’s protocol. One microgram of total DNA was used as the PCR template. The PCR reaction system and amplification cycle are shown in Additional file 1: Table S3. The PCR fragments of cytochrome B were purified and subjected to direct sequencing from both orientations using primers cytB-1 and cytB-2. The PCR products of MHC-DRB were recovered using an Agarose Gel DNA Purification Kit (OMEGA, U.S.A.), and cloned into pMD18-T vector (TaKaRa, Dalian, China). Ten colonies of each sample were selected and subjected to sequencing.

### Sequence analysis

All sequences were sorted with Clustalx software. Nucleotide variation sites were identified using MEGA5 software. The neighbor-joining phylogenetic tree was conducted based on Kimira two parameters using MEGA5 software. Bootstrap test value in the various branches of phylogenetic trees was obtained by repeating 1000 times, and all were considered as unordered sequence variation characteristics, DNA sequence variation in the transitions and transversions were given the same weight value.

### Statistical analysis

Statistical analyses were performed using the SPSS18.0 (IBM Corporation, Somers, NY, USA). The Fisher’s exact test was used to assess the statistical significance of differences. *P* < 0.05 was considered statistically significant.

## Results

### Chinese woodchucks from different areas have different susceptibility to WHV infection

Twelve animals each were captured in the TR, WL, TD, and GD counties, located in Qinghai province of China. Previous exposure to WHV was excluded in all animals by testing WHcAb, WHsAg, WHsAb, and WHV DNA. The age and the body weight of the animals from the 4 areas didn’t have significant differences (*P* = 0.179 and 0.094) (Table 1). All animals belonged to *M. himalayana* based on the morphological characteristics (Additional file 1: Table S1).

**Table 1:** Basic information

Area	n.	Characteristics		
		Gender (M/F)	Age* (M ± SD)	Body weight* (M ± SD)
TR	12	8/4	1.50 ± 1.04	4.70 ± 1.90
TD	12	4/8	2.00 ± 0.52	5.01 ± 0.89
GD	12	6/6	2.25 ± 0.26	6.13 ± 0.85
WL	12	5/7	2.08 ± 0.51	5.50 ± 1.75

Note: *P > 0.05

Twelve animals each were captured in the TR, WL, TD, and GD counties, located in Qinghai province of China. The age and the body weight of the animals from the 4 areas didn’t have significant differences

To analyze the susceptibility to WHV infection in animals from the 4 areas, 4 animals from each area were randomly selected, and routinely inoculated with the same dose of WHV59 stock. Afterwards WHV DNA and WHsAg were measured in serum every two weeks, and WHcAb was detected at the end of follow up.

**Fig. 1:**
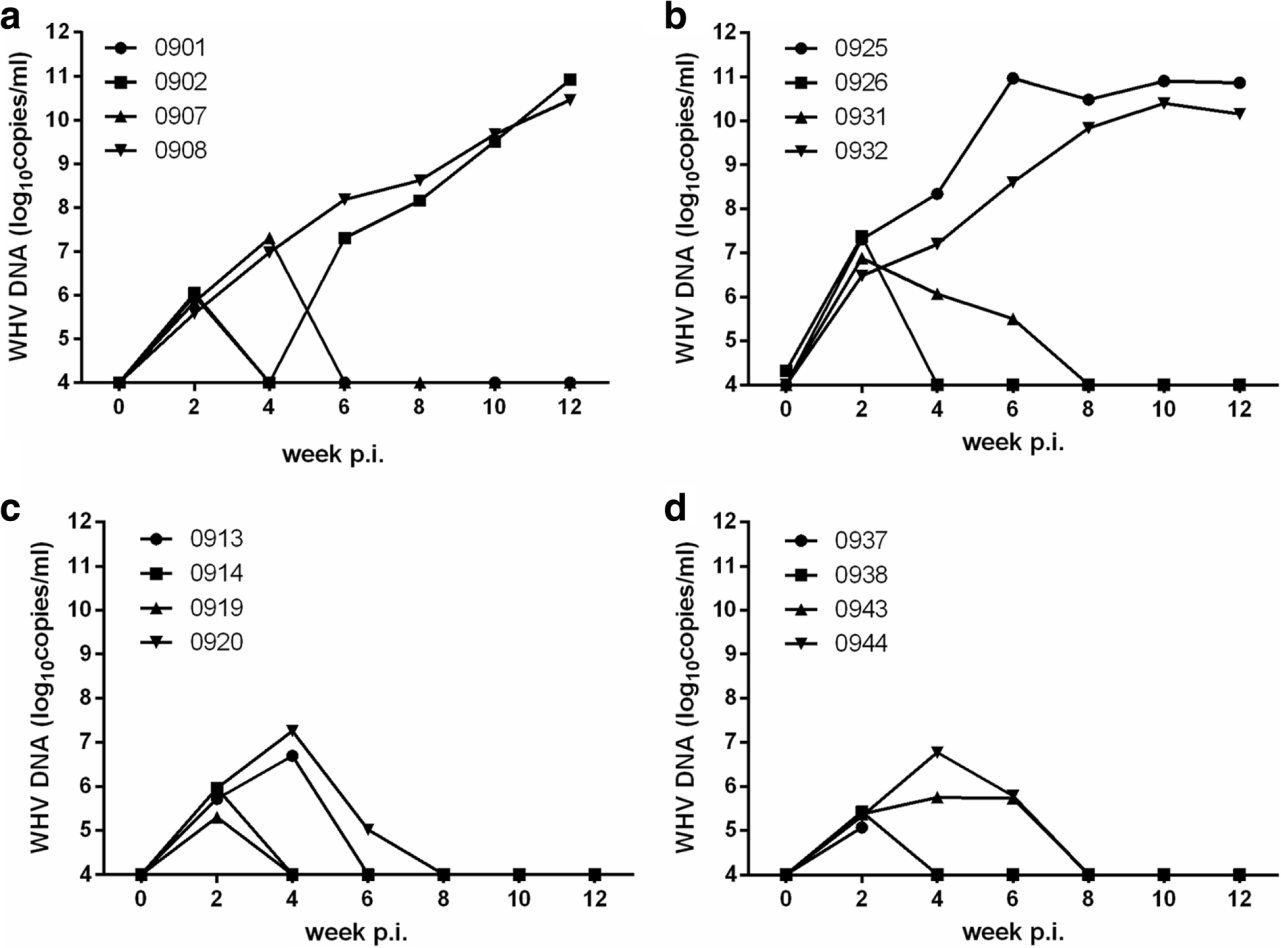
Kinetics of WHV DNA in WHV-infected Chinese woodchucks from the TR (**a**), TD (**b**), GD (**c**), and WL (**d**) areas in Qinghai province of China. Four animals from each area were randomly selected, and were inoculated with WHV59 strains. WHV DNA in serum was measured by real-time PCR at one-week interval

**Fig. 2:**
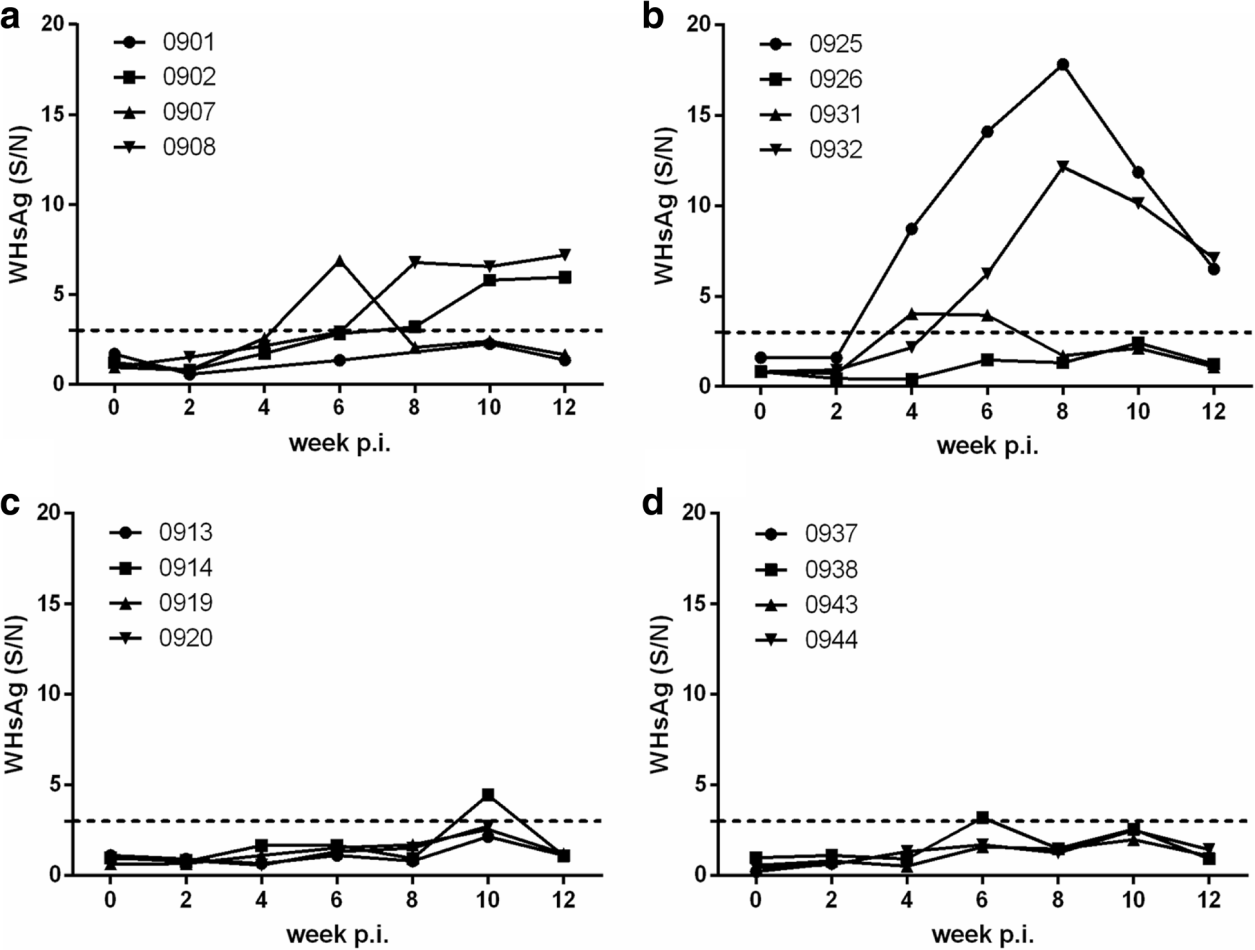
Kinetics of WHsAg in WHV-infected Chinese woodchucks from the TR (**a**), TD (**b**), GD (**c**), and WL (**d**) areas in Qinghai province of China. Four animals from each area were randomly selected, and were inoculated with WHV59 strains. WHsAg in serum was measured by ELISA at one-week interval. The results for WHsAg are presented as S/N values = OD of sample / OD of negative control. The cut-off value is presented by a dotted horizontal line

**Fig. 3:**
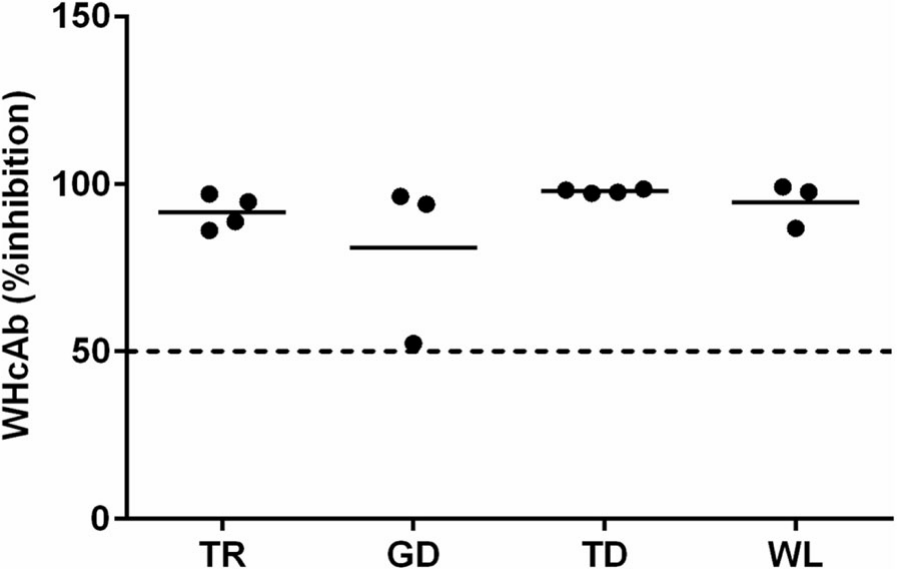
WHcAb in WHV-infected Chinese woodchucks from the TR, TD, GD, and WL areas in Qinghai province of China. Four animals from each area were randomly selected, and were inoculated with WHV59 strains. WHcAb in serum was measured by ELISA at the end of follow up (12 weeks post WHV inoculation). The results for WHcAb are presented as the percentage of inhibition = [((OD of negative control – OD of sample) / OD of negative control) × 100]. The cut-off value is 50%, and presented by a dotted horizontal line

WHV DNA appeared at a relatively low level of 10^5–7^ copies/ml in the sera of all animals at 2 weeks after WHV inoculation, but disappeared rapidly in all except animal 0920 (which died accidently at week 4) from the GD and WL area (Fig. 1C and D) and also in two animals each, from the TR and TD area (Fig.1A and 1B). In contrast, the level of WHV DNA in sera of the remaining animals from TR and TD areas gradually increased, and achieved high viral load of 10^10–11^ copies/ml (Fig. 1a and b). Serum WHsAg was negative in animals from GD and WL areas, while it was positive in 3 of 4 animals each from TR and TD areas (Fig. 2). WHcAb were positive at week12 in all animals from the 4 areas, except for 2 animals (0920 and 0937), which died accidentally at week 10 and week 4 post WHV inoculation (Fig. 3). These results indicated that Chinese woodchucks from different areas had different susceptibility to WHV infection.

### Chinese woodchucks from the 4 areas belong to the same subspecies *M. Himalayan robusta*

Whole blood was collected before WHV inoculation, and cytochrome B gene fragments were amplified using total DNA and the primer cytB-1 and cytB-2. The PCR products were directly sequenced, and cytochrome B sequences from 37 Chinese woodchucks were obtained, 11 from TR area, 8 from TD area, 9 from GD area, and 9 from WL area. Notably, only partial sequence of cytochrome B gene (nt 64–1080) was included for the analysis, since the direct sequencing only generated reliable data for the middle part of PCR fragments.

**Fig. 4:**
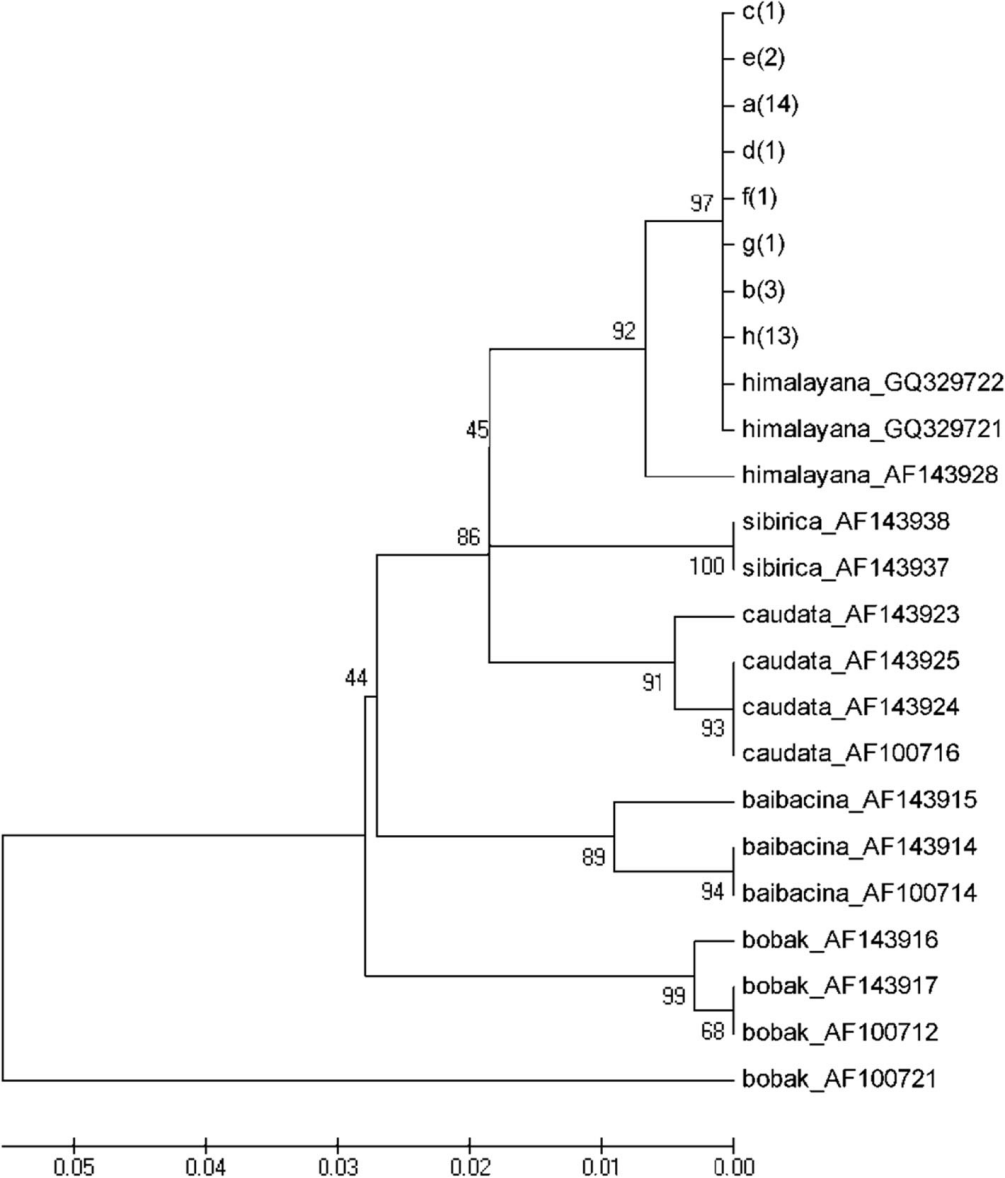
The neighbor-joining phylogenetic tree of the Chinese woodchuck species. The published cytochrome B sequences of the Chinese woodchuck species (*M. himalayana*, *M. sibirica*, *M. caudata*, *M. baibacina*, and *M. bobak)* were downloaded at least two sequences for one species. The 8 cytochrome B alleles were obtained in 37 animals from TR, TD, GD, and WL areas, Qinghai province, China. The neighbor-joining phylogenetic tree was conducted using MEGA5 software

All of the sequences were sorted by Clustalx software, and the nucleotide substitutions at nt 141, nt 186, nt 256, nt 331, nt 349, nt 571, nt 676, and nt 695 were identified. Based on the pattern of the substitutions, all of the sequences were assigned to 8 alleles, named A, B, C, D, E, F, G, and H (Table 2). The distribution of the 8 alleles were listed in Additional file 1: Table S4. The 8 alleles were submitted to GenBank (accession numbers: KF996568-KF996575). Chinese woodchucks mainly belong to 5 species, *M. himalayana*, *M. sibirica*, *M. caudata*, *M. baibacina*, and *M. bobak*, therefore the cytochrome B sequences of the 5 Chinese woodchuck species were downloaded from GenBank, at least two sequences for each species. The phylogenetic tree of cytochrome B sequences was conducted using the 14 downloaded sequences and the 8 alleles, and indicated that all of the animals from the 4 areas belonged to the same species *M. himalayana* (Fig. 4).

**Table 2:** Nucleotide substitutions in the 8 mitochondrial cytochrome B alleles from *M. himalayana*

Nucleotide position	Consensus sequence	Alleles							
		A	B	C	D	E	F	G	H
141	C		T						
186	C			T					
256	G				A				
331	G					A			
349	G						A		
571	G							A	
676	A					G			
695	T			C	C		C		C

The nucleotide substitutions at nt 141, nt 186, nt 256, nt 331, nt 349, nt 571, nt 676, and nt 695 were identified. Based on the pattern of the substitutions, all of the sequences were assigned to 8 alleles, named A, B, C, D, E, F, G, and H

Three cytochrome B sequences of *M. himalayana* were previously published in GenBank. One sequence (AF143928) from *M. himalayana robusta* was published by Steppen et al., and the animals were collected from Yushu, Qinghai, China. The other two (GQ329721 and GQ329722) were published by Zhang et al., and there was no information about the location and the subspecies of the animals used to obtain the sequences. The intraspecific phylogenetic tree of *M. himalayana* was conducted using the 2 published sequences (AF143928 and GQ329721) and the 8 obtained alleles, suggesting all of the animals from the 4 areas belonged to the same subspecies *M. himalayana robusta* (Fig. 4). We also calculated the genetic distance among *M. himalayana* using the 2 published sequences and the 8 obtained alleles of cytochrome B, and found the genetic distances varied from 0.000 to 0.009 (Table 3), suggesting all of the animals used in this study, Xining isolates, and *M. himalayana robusta* are closely related, and belong to the same subspecies.

**Table 3:** Genetic distances of the 8 mitochondrial cytochrome B alleles from *M. himalayana*

Allele	Allele								Xining	Yushu
	A	B	C	D	E	F	G	H		
A		0.000	0.000	0.003	0.004	0.003	0.003	0.000	0.000	0.006
B	0.000		0.000	0.003	0.004	0.003	0.003	0.000	0.000	0.006
C	0.000	0.000		0.003	0.004	0.003	0.003	0.000	0.000	0.006
D	0.003	0.003	0.003		0.005	0.004	0.004	0.003	0.003	0.007
E	0.006	0.006	0.006	0.009		0.005	0.005	0.004	0.004	0.007
F	0.003	0.003	0.003	0.006	0.009		0.004	0.003	0.003	0.007
G	0.003	0.003	0.003	0.006	0.009	0.006		0.003	0.003	0.007
H	0.000	0.000	0.000	0.003	0.006	0.003	0.003		0.000	0.006
Xining*	0.000	0.000	0.000	0.003	0.006	0.003	0.003	0.000		0.012
Yushu**	0.012	0.012	0.012	0.015	0.018	0.015	0.015	0.012	0.006	

Estimates of the genetic distances and standard errors are presented above and below the diagonal, respectively

*The accession number of cytochrome B gene sequences of *M. himalayana* Xining isolates is GQ329721

**The accession number of cytochrome B gene sequences of *M. himalayana* from Yushu, Qinghai, China is AF143928

The genetic distance among *M. himalayana* was calculated using the 2 published sequences(*M. himalayana* from Xining GQ329721 and *M. himalayana* from Yushu AF143928) and the 8 obtained alleles of cytochrome B

### The genetic variation of mitochondrial cytochrome B differed in *M. himalayana* with the different susceptibility to WHV infection

Since the mitochondrial DNA haplotype frequencies were considered to be primarily controlled by migration and genetic drift, and that most intraspecies variation is selectively neutral, we compared the frequencies of the 8 cytochrome B alleles in the animals from the four areas (Table 4). Most of the animals (72.97%, 27/37) have allele A (37.84%, 14/37) or H (35.14%, 13/37), indicating these two alleles were the predominant alleles in the animals from TR, TD, GD, and WL areas of Qinghai province (Table 4). Allele A is the consensus allele, while allele H has a nucleotide substitution at nt 695 from T to C (Table 2). Based on the different susceptibility to WHV infection, the animals from the four different areas could be divided into two subgroups, those from TR and TD areas belonged to one subgroup which had the high replicative pattern, while those from GD and WL areas belonged to the other and had the low replicative pattern of WHV infection. To define the relationship of the two predominant alleles and the two replicative pattern of WHV infection, we compared the frequencies of the two alleles in the two subgroups.

**Table 4:** The frequencies of the 8 cytochrome B alleles in *M. himalayana* from the 4 areas with different susceptibility to WHV infection

WHV susceptibility	Area	n.	The frequency of the alleles (%)							
			A	B	C	D	E	F	G	H
High replication pattern	TR	11	72.73 (8/11)	0 /	0 /	9.09 (1/11)	18.18 (2/11)	0 /	0 /	0 /
	TD	9	44.44 (4/9)	33.33 (3/9)	0 /	0 /	0 /	0 /	11.11 (1/9)	11.11 (1/9)
	Total	20	60.00 (12/20)	15.00 (3/20)	/ /	5.00 (1/20)	10.00 (2/20)	0 /	5.00 (1/20)	5.00 (1/20)
Low replication pattern	GD	9	22.22 (2/9)	11.11 (1/9)	11.11 (1/9)	0 /	0 /	0 /	0 /	55.55 (5/9)
	WL	8	0 /	0 /	0 /	0 /	0 /	12.5 (1/8)	0 /	87.5 (7/8)
	Total	17	11.76 (2/17)	5.88 (1/17)	5.88 (1/17)	0 /	0 /	5.88 (1/17)	0 /	70.59 (12/17)
Total		37	37.84 (14/37)	10.81 (4/37)	2.70 (1/37)	2.70 (1/37)	5.4 (2/37)	2.70 (1/37)	2.70 (1/37)	35.14 (13/37)

Cytochrome B sequences from 37 Chinese woodchucks were obtained, 11 from TR area, 8 from TD area, 9 from GD area, and 9 from WL area. The frequencies of the 8 cytochrome B alleles in the animals from the four areas were calculated

The frequencies of allele A in the high and low replicative subgroups were 60.00% (12/20) and 11.76% (2/17) (*P* = 0.1089), respectively, while those of allele H were 5.00% (1/20) and 70.59% (12/17) (*P* < 0.0001), respectively (Table 4). These results indicated allele A and H of mitochondrial cytochrome B gene may be predominant in animals with the high or low replicative pattern of WHV infection, respectively.

### The genetic variation of MHC-DRB differed in *M. himalayana* with different susceptibility to WHV infection

**Fig. 5:**
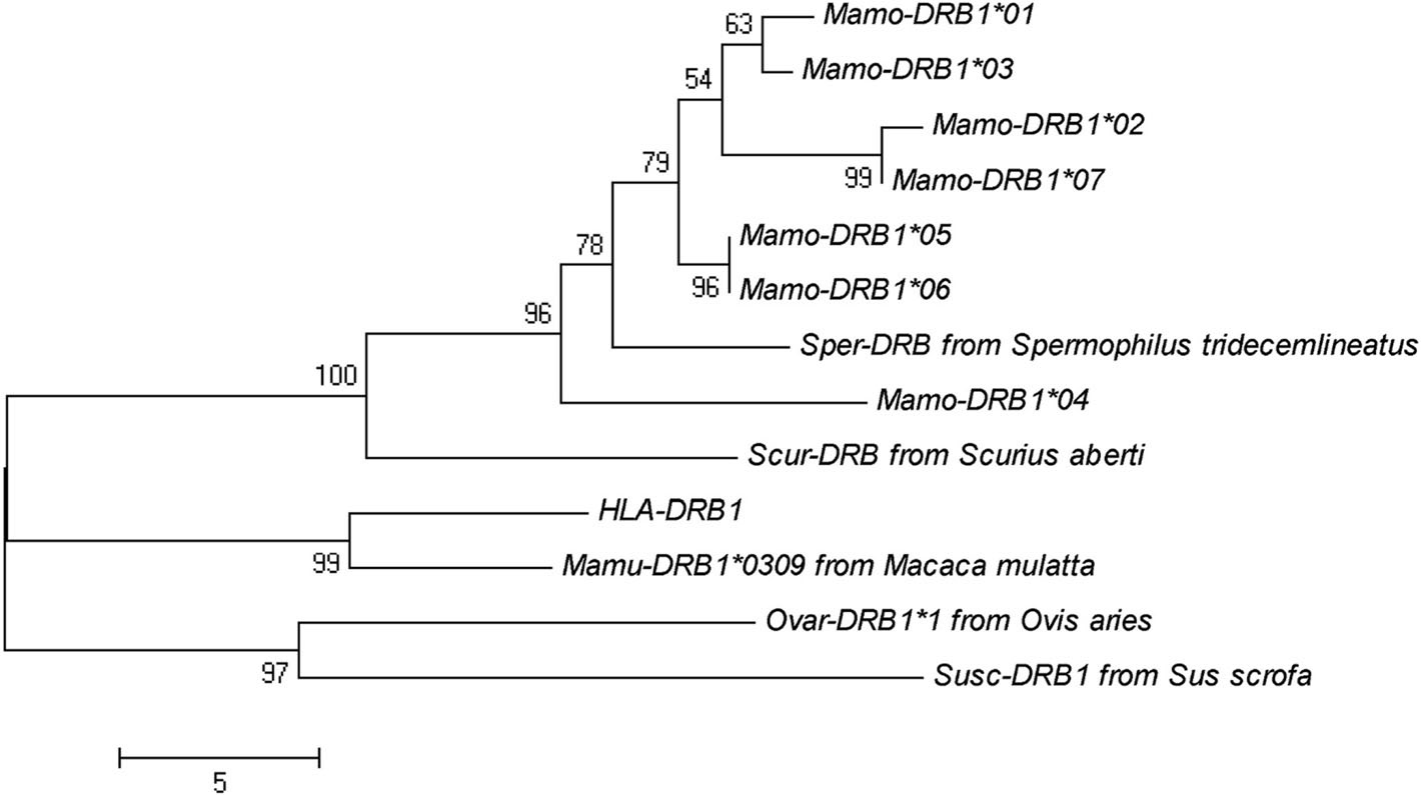
A phylogenetic tree constructed using 789 bp fragments of MHC class II DRB1 genes from Chinese woodchucks. The sequences used in constructing the phylogenetic tree are as following: Sper-DRB from *Spermophilus tridecemlineatus* (XM005338933), Scur-DRB from *Sciurus aberti aberti* (M97616.1), Mamu-DRB1 from *Macaca mulatta* (EF362437.1), Ovar-DRB1 from *Ovis aries* (KM588646.1), Susc-DRB1 from *Sus scrofa* (EU431221.1), HLA-DRB1 from *Homo sapiens* (M33600.1)

**Fig. 6:**
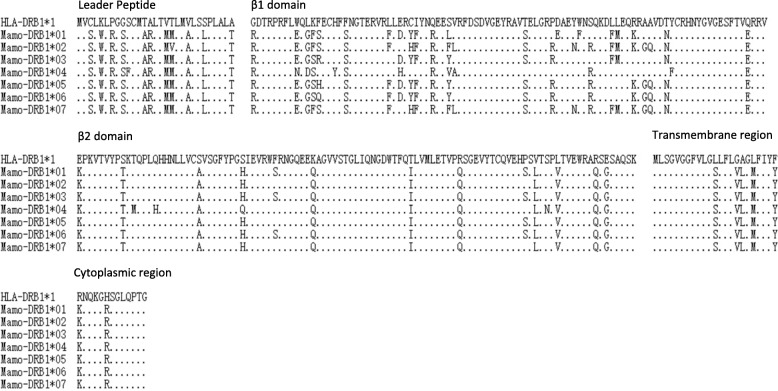
Deduced amino acid sequences of partial Mamo-DRB1 alleles. The HLA-DRB1*1 sequence is chosen as reference. Identity to the consensus is illustrated by spots

Whole blood was collected before WHV inoculation, and MHC-DRB gene fragments were amplified using total RNA and the primers DRB-1 and DRB-2. The PCR products were cloned into pMD18-T, and the positive clones were sequencing. The partial sequences of MHC-DRB molecules were obtained from 19 animals, including 9 animals from TR area, 2 from TD area, 3 from GD area, and 5 from WL area. All of the sequences were sorted by MEGA software, and 7 alleles were identified. Phylogenetic analysis indicated that they are closely related to MHC-DRB genes from *Spermophilus tridecemlineatus* and *Sciurus aberti aberti*, and MHC-DRB1 genes from human (Fig. 5). Therefore, these 7 alleles were named as MhcMamo-DRB1*01, MhcMamo-DRB1*02, MhcMamo-DRB1*03, MhcMamo-DRB1*04, MhcMamo-DRB1*05, MhcMamo-DRB1*06, and MhcMamo-DRB1*07 [21]. The alignment of the 7 alleles from *M. himalayana* indicated that the amino acid variation mainly occurred in the β1 domain, in which 24 amino acid sites were not conserved. These are consistent with the previous results from *Marmota monax* [22] and rhesus macaque [23]. While the transmembrane region and the cytoplasmic region are conserved (Fig. 6).

The distribution and the frequencies of MHC-DRB alleles in animals from different areas were listed in Additional file 1: Table S5 and Table 5. At least 5 of 19 animals were heterozygous, while we obtained only one allele in the other 14 animals. Most of the animals (84.21%, 16/19) have Mamo-DRB1*02 (57.89%, 11/19) or Mamo-DRB1*07 (26.32%, 5/19), indicating these two alleles were the predominant alleles in the animals from TR, TD, GD, and WL areas of Qinghai province (Table 5).

**Table 5:** The frequencies of the 7 MHC-DRB1 alleles in *M. himalayana* from the 4 areas with different susceptibility to WHV infection

WHV susceptibility	Area	n.	The frequency of the *Mamo-DRB1* alleles (%)						
			*01	*02	*03	*04	*05	*06	*07
High replication pattern	TR	9	22.22 (2/9)	77.78 (7/9)	11.11 (1/9)	11.11 (1/9)	11.11 (1/9)	22.22 (2/9)	/
	TD	2	/	50.00 (1/2)	/	/	/	/	50.00 (1/2)
	Total	11	18.18 (2/11)	72.73 (8/11)	9.09 (1/11)		9.09 (1/11)	18.18 (1/11)	9.09 (1/11)
Low replication pattern	GD	3	/	33.33 1/3	/	/	/	33.33 (1/3)	66.66 (2/3)
	WL	5	/	40.44 2/5	/	/	20.00 1/5	/	40.00 (2/5)
	Total	8	/	37.50 3/8	/	/	12.5 1/8	12.5 (1/8)	50.00 (4/8)
Total		19	10.53 2/19	57.89 11/19	5.26 1/19	5.26 1/19	10.53 2/19	10.53 2/19	26.32 5/19

The MHC class II DRB1 sequences from 19 Chinese woodchucks were obtained, 9 from TR area, 2 from TD area, 3 from GD area, and 5 from WL area. The frequencies of the 7 MHC-DRB1 alleles in the animals from the four areas were compared

As many associations between MHC-DRB alleles and susceptibility to infectious and autoimmune diseases have been described in humans or in rodent [19, 24, 25], we compared the frequencies of the two alleles in the animals with different susceptibility to WHV infection. The frequencies of Mamo-DRB1*02 in the high and low replicative subgroups were 72.73% (8/11) and 37.5% (3/8) (*P* = 0.4661), respectively, while those of Mamo-DRB1*07 were 9.09% (1/11) and 50% (4/8) (*P* = 0.1108), respectively (Table 5). These results indicated the two alleles Mamo-DRB1*02 and Mamo-DRB1*07 may be predominant in animals with the high or low replicative pattern of WHV infection, respectively.

## Discussion

In this study, we found that the animals from four different areas had different susceptibility to WHV infection. Animals from TR and TD areas had a high level of long-lasting viremia, while those from GD and WL areas had a low level of transient viremia after WHV inoculation. All of the animals belong to the same subspecies *M. himalayana robusta* identified by the analysis of mitochondrial cytochrome B sequences. Based on the profiles of the nucleotide substitutions, eight different alleles of mitochondrial cytochrome B gene were identified in the animals used in this study. Two alleles, allele A (the consensus sequence) and H (nucleotide substitution at nt 695 from T to C) predominate. Allele A was frequent in animals from TR and TD areas (60.00%) which had the high replicative pattern of WHV infection. Allele H was predominant in animals from GD and WL areas (70.59%) which had the low replicative pattern. Based on the profiles of the nucleotide substitutions, 7 MHC-DRB alleles were identified in Chinese woodchucks from 4 different areas, and are closely related to the counterparts of *Spermophilus tridecemlineatus, Sciurus aberti aberti*, and human. Two alleles, Mamo-DRB1*02 and Mamo-DRB1*07 predominate. Allele Mamo-DRB1*02 was frequent in animals from TR and TD areas (72.73%) which had the high replicative pattern of WHV infection. Allele Mamo-DRB1*07 was predominant in animals from GD and WL areas (50.00%) which had the low replicative pattern.

Chinese woodchucks (*M. himalayana*) from different areas had different susceptibility to the experimental WHV infection, those from TR and TD areas had a high level of long-lasting viremia, while those from GD and WL areas had a low level of transient viremia. This is consistent with the previous study using American woodchucks (*M. monax*). Animals from mid-Atlantic states had higher frequencies of naturally occurring WHV infection then those from New York and much of New England [13]. Consistently, there is considerable heterogeneity in susceptibility to virus infection among exposed human, e.g. HBV [26], hepatitis C virus (HCV) [25], and human immunodeficiency virus (HIV) [27].

Genetic variation was already found in the American woodchuck populations with high and low prevalence of naturally occurring WHV infection [13]. Wright et al. identified a significant heterogeneity of plasma proteins and erythrocyte enzymes between the two populations using starch gel electrophoresis. In our study, genetic variation of mitochondrial cytochrome B was found in the two Chinese woodchuck populations with different outcomes of experimental WHV infection. Though all of the animals belong to the same subspecies *M. himalayana, robusta*, cytochrome B Allele A was predominant in animals with high replicative pattern of WHV infection, while Allele H (nucleotide substitution at nt 695 from T to C) in those with low replicative pattern. These results indicated that genetic variation may be associated with susceptibility not only to naturally occurring but also to experimentally WHV infection in woodchucks. Furthermore, cytochrome B Alleles A and H will be of benefit for the selection of high WHV susceptible Chinese woodchucks, which could be used as an animal model to evaluate antiviral drugs or therapeutic vaccines [20, 28].

Host genetic background was found to contribute to viral susceptibility, e.g. single nucleotide polymorphism in IL28B strongly predicted spontaneous HCV recovery [29] and the C-C chemokine receptor (CCR5) was identified to restrict HIV infection [30]. MHC restricted CD4 and CD8 T cell responses play an important role in determining the outcome of HBV infection [31, 32]. In our study, we analyzed MHC-DRB sequences in woodchucks with different susceptibility to WHV infection. We identified 7 MHC-DRB alleles from *M. himalayana* for the first time, in which alleles Mamo-DRB1*02 and Mamo-DRB1*07 are predominate. Furthermore, Allele Mamo-DRB1*02 was frequent in animals from TR and TD areas which had the high replicative pattern of WHV infection. While allele Mamo-DRB1*07 was frequent in animals from GD and WL areas which had the low replicative pattern. This is consistent with the previous study in human. HLA-DRB1*12 carriers may have a high risk for HBV infection, but DRB1*0712 may be implied in viral persistence. HLA-DRB1*11 and 14 may be associated with viral clearance in the cases of HBV subgenotype C2 infection [19]. The association between virus susceptibility or disease severity and MHC alleles may be related with the unequal T cell response restricted by different MHC alleles. Individuals with HLA-B*3501 have an increased risk of developing severe Hantavirus pulmonary syndrome (HPS), and have significantly higher frequencies of SNV-specific HLA-B*3501 restricted T cells [33]. Individuals with the haplotype HLA-B*08-HLA-DRB1*0301 are prone to have normal or increased humoral immune response and a low T-cell immune responsiveness [34]. Furthermore, Mamo-DRB1*02 and Mamo-DRB1*07 alleles will be of benefit for the selection of high WHV susceptible Chinese woodchucks, which could be used as an animal model to evaluate antiviral drugs or therapeutic vaccines.

## Conclusions

Chinese woodchucks from TR and TD areas had high and long-lasting viremia, while those from WL and GD areas had low and short-lasting viremia, though they belong to the same subspecies. The genetic variation of cytochrome B and MHC-DRB differed in Chinese woodchucks with different susceptibility to WHV infection. These data will be benefit for understanding the pathogenesis of hepadnavirus infection, and cytochrome B and MHC-DRB alleles will be helpful for selection suitable animals for WHV infection.

## Additional file

Additional file 1: **Table S1.** Basic information. **Table S2.** Primers used for PCR and RT-PCR. **Table S3.** The parameters of PCR reaction mix and the amplification cycles. **Table S4.** Distribution of cytochrome B alleles in Chinese woodchucks. **Table S5.** Distribution of MHC-DRB alleles in Chinese woodchucks. (ZIP 41 kb)
