# Supplementary material for: Chinese woodchucks with different susceptibility to WHV infection differ in their genetic background exemplified by cytochrome B and MHC-DRB molecules
Source: Virol J. 2018 Jun 18;15:101. doi: 10.1186/s12985-018-1010-y (PMC6006932; doi:10.1186/s12985-018-1010-y)
Supplement: Supplementary file 1 — Table S1. Basic information. Table S2. Primers used for PCR and RT-PCR. Table S3. The parameters of PCR reaction mix and the amplification cycles. Table S4. Distribution of cytochrome B alleles in Chinese woodchucks. Table S5. Distribution of MHC-DRB alleles in Chinese woodchucks. (ZIP 41 kb) [file 12985_2018_1010_MOESM1_ESM.zip]

**Table S1. Basic information**

| **Area** | **No.** | **Gender*** | **Age†** | **Body weight** | **Species^#^** |
| --- | --- | --- | --- | --- | --- |
| TR | **0901^＆^** | **F** | **0.5** | **2.1** | ***M．himalayana*** |
|  | **0902^＆^** | **M** | **0.5** | **3.8** | ***M．himalayana*** |
|  | 0903 | M | 0.5 | 3.3 | *M．himalayana* |
|  | 0904 | M | 0.5 | 2.9 | *M．himalayana* |
|  | 0905 | F | 0.5 | 2.4 | *M．himalayana* |
|  | 0906 | F | 0.5 | 3.5 | *M．himalayana* |
|  | **0907^＆^** | **F** | **2.5** | **4.9** | ***M．himalayana*** |
|  | **0908^＆^** | **M** | **2.5** | **6.5** | ***M．himalayana*** |
|  | 0909 | M | 2.5 | 7.1 | *M．himalayana* |
|  | 0910 | M | 2.5 | 6.6 | *M．himalayana* |
|  | 0911 | M | 2.5 | 6.5 | *M．himalayana* |
|  | 0912 | M | 2.5 | 6.8 | *M．himalayana* |
| GD | **0913^＆^** | **F** | **1.5** | **4.3** | ***M．himalayana*** |
|  | **0914^＆^** | **F** | **1.5** | **3.4** | ***M．himalayana*** |
|  | 0915 | M | 1.5 | 4.7 | *M．himalayana* |
|  | 0916 | F | 1.5 | 4.4 | *M．himalayana* |
|  | 0917 | F | 1.5 | 4.6 | *M．himalayana* |
|  | 0918 | F | 1.5 | 4.7 | *M．himalayana* |
|  | **0919^＆^** | **F** | **2.5** | **6.4** | ***M．himalayana*** |
|  | **0920^＆^** | **F** | **2.5** | **5.5** | ***M．himalayana*** |
|  | 0921 | M | 2.5 | 6.6 | *M．himalayana* |
|  | 0922 | F | 2.5 | 5 | *M．himalayana* |
|  | 0923 | M | 2.5 | 5.2 | *M．himalayana* |
|  | 0924 | M | 2.5 | 5.3 | *M．himalayana* |
| TD | **0925^＆^** | **M** | **2.0** | **6.6** | ***M．himalayana*** |
|  | **0926^＆^** | **M** | **2.0** | **6.1** | ***M．himalayana*** |
|  | 0927 | M | 2.0 | 6.8 | *M．himalayana* |
|  | 0928 | M | 2.0 | 6.2 | *M．himalayana* |
|  | 0929 | F | 2.0 | 6.3 | *M．himalayana* |
|  | 0930 | F | 2.0 | 6.4 | *M．himalayana* |
|  | **0931^＆^** | **F** | **2.5** | **5.4** | ***M．himalayana*** |
|  | **0932^＆^** | **F** | **2.5** | **5** | ***M．himalayana*** |
|  | 0933 | F | 2.5 | 5.1 | *M．himalayana* |
|  | 0934 | F | 2.5 | 5 | *M．himalayana* |
|  | 0935 | M | 2.5 | 7.7 | *M．himalayana* |
|  | 0936 | M | 2.5 | 6.9 | *M．himalayana* |
| WL | **0937^＆^** | **F** | **1.5** | **3.9** | ***M．himalayana*** |
|  | **0938^＆^** | **F** | **1.5** | **3.9** | ***M．himalayana*** |
|  | 0939 | F | 1.5 | 3.8 | *M．himalayana* |
|  | 0940 | F | 1.5 | 3.9 | *M．himalayana* |
|  | 0941 | F | 1.5 | 3.9 | *M．himalayana* |
|  | 0942 | M | 2.5 | 8.3 | *M．himalayana* |
|  | **0943^＆^** | **F** | **2.5** | **7.9** | ***M．himalayana*** |
|  | **0944^＆^** | **M** | **2.5** | **5.3** | ***M．himalayana*** |
|  | 0945 | M | 2.5 | 8.1 | *M．himalayana* |
|  | 0946 | M | 2.5 | 5.7 | *M．himalayana* |
|  | 0947 | M | 2.5 | 5.8 | *M．himalayana* |
|  | 0948 | F | 2.5 | 5.5 | *M．himalayana* |

^*^: Identification of gender was based on the characteristic of genitalia

^†^: Identification of age was based on the zoological characteristics of the body including the tooth, the tail, and the genitalia, etc.

**^#^**: Identification of species was based on the morphological characteristics

**^＆^**: These animals were used for experimental WHV infection

**Table S2. Primers used for PCR and RT-PCR**

| Name | Orientation | Nucleotide sequences | nt positions |
| --- | --- | --- | --- |
| CYT-1 | sense | 5'ATGACAAACATCCGCA 3' | 1-16 |
| CYT-2 | anti-sense | 5'TCTTCATTTAAGAAGTTTGT 3' | 1140-1121 |
| DRB-1 | sense | 5'ATGGTGAGTCTGTGGC 3' | 1-16 |
| DRB-2 | anti-sense | 5'TCCTGTTGGCTGAAGTCC 3' | 772-789 |

**Table S3. The parameters of PCR reaction mix and the amplification cycles**

| Ingredient | Concentration | Volume (µL) /reaction |
| --- | --- | --- |
| RNA template | 0.5ug/µL | 2.0 |
| Oligo(dT)_15_ | 500ug/mL | 1.5 |
| 70℃ 5min for denaturation，quick-cooling on ice，short centrifugation | | |
| 5×buffer |  | 5.0 |
| dNTPs | 10mmol/L | 1.5 |
| Rnasin | 50U/µL | 0.5 |
| M-MLV revertase | 200U/µL | 1.0 |
| ddH_2_O |  | 13.5 |
| Total volume |  | 25 |

| Ingredient | Concentration | Volume (µL) /reaction |
| --- | --- | --- |
| 10×reaction buffer (Mg^2+^) |  | 5.0 |
| dNTPs | 10mmol/L | 1.0 |
| P1 | 10umol/lL | 1.0 |
| P2 | 10mmol/L | 1.0 |
| Taq polymerase | 5U/µL | 1.0 |
| cDNA template |  | 2.0 |
| ddH_2_O |  | 39 |
| Total volume |  | 50 |

| PCR cycle conditions | [temperature](file:///F:\%E7%A8%8B%E5%BA%8F%E8%BD%AF%E4%BB%B6\%E6%9C%89%E9%81%93%E8%AF%8D%E5%85%B8\Dict\7.0.0.1203\resultui\dict\result.html?keyword=temperature)（℃） | Time（minutes） |
| --- | --- | --- |
| Initial denaturation | 95 | 5.0 |
| denaturation^*^ | 94 | 1.0 |
| annealing^*^ | 53 | 1.0 |
| extension^*^ | 72 | 1.0 |
| Ultimate extension | 72 | 7.0 |

^*^: Major amplification cycle was 30 times

**Table S4. Distribution of cytochrome B alleles in Chinese woodchucks**

| **Alleles** | **Area** | **Animal No.** |
| --- | --- | --- |
| A | TR | **0901***, 0904, 0905, 0906, **0907***, **0908***, 0910, 0911 |
|  | TD | **0926***, 0930, 0935, 0936 |
|  | GD | 0921, 0922 |
|  | WL | **/** |
| B | TR | **/** |
|  | TD | 0928, 0929, 0933 |
|  | GD | **0920*** |
|  | WL | **/** |
| C | TR | **/** |
|  | TD | **/** |
|  | GD | 0924 |
|  | WL | **/** |
| D | TR | 0902 |
|  | TD | **/** |
|  | GD | **/** |
|  | WL | **/** |
| E | TR | 0909, 0912 |
|  | TD | **/** |
|  | GD | **/** |
|  | WL | **/** |
| F | TR | **/** |
|  | TD | **/** |
|  | GD | **/** |
|  | WL | **0937*** |
| G | TR | **/** |
|  | TD | 0927 |
|  | GD | **/** |
|  | WL | **/** |
| H | TR | / |
|  | TD | **0925*** |
|  | GD | 0916, 0917, 0918, 0919, 0923 |
|  | WL | 0939, 0940, 0941, 0942, **0943*** |

**^*^**: These animals were used for experimental WHV infection

**Table S5. Distribution of MHC-DRB alleles in Chinese woodchucks**

| **Area** | **No.** | **Alleles (number of obtained sequences)** |
| --- | --- | --- |
| TR | **0901^†^** | *Mamo-DRB1*01*(2)/ *Mamo-DRB1*02*(1) |
|  | 0903 | *Mamo-DRB1*02*(1) |
|  | 0904 | *Mamo-DRB1*02*(2)/ *Mamo-DRB1*03*(3) |
|  | 0906 | *Mamo-DRB1*01*(1)/ *Mamo-DRB1*02*(3) |
|  | **0907^†^** | *Mamo-DRB1*02*(3) |
|  | **0908^†^** | *Mamo-DRB1*04*(3) |
|  | 0910 | *Mamo-DRB1*05*(3) |
|  | 0911 | *Mamo-DRB1*02*(1)/ *Mamo-DRB1*06* (1) |
|  | 0912 | *Mamo-DRB1*02*(5) |
| TD | **0932^†^** | *Mamo-DRB1*07*(1) |
|  | 0936 | *Mamo-DRB1*02*(7) |
| GD | **0913^†^** | *Mamo-DRB1*02*(8) |
|  | 0916 | *Mamo-DRB1*06*(1)/ *Mamo-DRB1*07*(1) |
|  | 0923 | *Mamo-DRB1*07*(4) |
| WL | 0940 | *Mamo-DRB1*02*(4) |
|  | 0941 | *Mamo-DRB1*02*(10) |
|  | 0945 | *Mamo-DRB1*05*(9) |
|  | 0947 | *Mamo-DRB1*07*(2) |
|  | 0948 | *Mamo-DRB1*07*(1) |

**^†^**: These animals were used for experimental WHV infection
